# Mutational signatures of synchronous and metachronous brain metastases from lung adenocarcinoma

**DOI:** 10.1186/s40164-023-00418-x

**Published:** 2023-06-13

**Authors:** Jianing Chen, Hainan Yang, Chao Zhao, Tao Lin, Da Liu, Weiping Hong, Changguo Shan, Cheng Zhou, Ling Bao, Caicun Zhou, Linbo Cai, Chunxia Su, Zhaoming Zhou, Lei Wen

**Affiliations:** 1 Department of Medical Oncology, Shanghai Pulmonary Hospital & Thoracic Cancer Institute, Tongji University School of Medicine, Shanghai, People’s Republic of China; 2 Department of Neurosurgery, Guangdong Sanjiu Brain Hospital, Guangzhou, People’s Republic of China; 3 Department of Oncology, Guangdong Sanjiu Brain Hospital, Shatai North Road, Guangzhou, People’s Republic of China; 4 Department of Geriatrics, The First Affiliated Hospital of Bengbu Medical College, Bengbu, People’s Republic of China; 5 Clinical Research Center, Shanghai Pulmonary Hospital, 200433 Shanghai, People’s Republic of China

**Keywords:** Lung adenocarcinoma, Brain metastasis, Cerebrospinal fluid

## Abstract

Brain metastasis (BM) is an important cause of mortality for cancer patients. Many patients were diagnosed with brain metastases at their first visit who have not received any treatment while a subset of patients did not have distant metastases at the first visit and brain metastases were detected during the course of systemic therapies. The difference in their genomic characterization is unclear. 96 lung adenocarcinoma patients were enrolled in our study. 53 patients (55%) had synchronous metastatic brain tumors. 43 (45%) patients had metachronous brain metastases. We performed 168 panel-targeted gene sequencing cerebrospinal fluid (CSF) and plasma samples from patients to identify genomic features of synchronous brain metastases (SBM) and metachronous brain metastases (MBM). In conclusion, CSF liquid biopsies have a priority in detecting gene alteration. A comprehensive comparison of molecular profiling between SBM and MBM revealed the most frequently altered genes in both groups were *EGFR* and *TP53*, but with different exon point mutations. RTK-RAS and TP53 pathways were the most affected pathways.

**Supplementary Information:**

The online version contains supplementary material available at 10.1186/s40164-023-00418-x.

**To the Editor,**

Brain metastases (BM) are observed in 30% to 50% of patients at initial diagnosis with more patients developing metastases during treatment [1]. Synchronous brain metastases (SBM) and metachronous brain metastases (MBM) are two distinct clinical conditions. Understanding the intrinsic factors of discrepancy and developing therapeutic strategies is an urgent need. The immune-suppressive microenvironment of SBM and MBM has recently been investigated closely [2]. However, the mutational profiles of SBM and MBM are still uncharacterized. Herein, we outlined the mutational characteristics of SBM and MBM, and explored the molecular pathways to search for potential treatment strategies.

The clinical characteristic of patients diagnosed with BM was summarized in Additional file 6: Table S1. Fifty-three patients who had SBM at the first diagnosis were defined as the synchronous group. Forty-three MBM patients were in stage I to IV stage at the first diagnosis and then developed BM over the course of the disease. Long-term follow-up overall survival (OS) of two cohorts showed that patients in the synchronous group had an OS of 31 (95% CI 21.19–40.81) months and were shorter compared to patients in the metachronous group with an OS of 71 (95% CI 34.14–107.86) months (*p* = 0.0005) (Additional file 1: Figure S1). We further calculated the brain overall survival (BOS), defined as the time from the diagnosis of brain metastases to the end of follow-up. The median BOS was 31 (95% CI 21.19–40.81) months in the SBM group and 39 (95% CI 21.80–56.20) months in the MBM group, with no statistically significant difference between the two groups (P = 0.32) (Additional file 2: Figure S2). In MBM group, the median interval time of brain parenchymal metastases (BPM) and Leptomeningeal metastases (LM) from diagnosis was 21.3 months (IQR, 14.8 – 37.5) and 38.4 months (IQR, 19.6 – 56.0).

We have identified mutations in the plasma and CSF samples from ninety-six patients enrolled in our study. Figure 1 displayed the mutation profiles of the most frequently detected mutations from plasma (Fig. 1A) and CSF (Fig. 1B) samples. In our research, we have also analyzed the plasma samples of 11 patients with liver metastases (Additional file 3: Figure S3) and 29 patients with bone metastases (Additional file 4: Figure S4). The top 3 gene mutation were EGFR, TP53, and MYCN in 29 patients, EGFR TP53, GRIN2A were most frequently mutated in 11 patients with liver metastasis. Compared to blood plasma, CSF samples revealed a more comprehensive genomic characterization of BM. The detection rate of *EGFR* in CSF was 58% and in plasma was 32%. *TP53* mutation was detected in 48% and 13% of CSF and plasma, respectively. According to the subtypes of *EGFR*, alterations were more detected in CSF while they were less detected or not available in plasma samples (Fig. 1C). Within the individual patient, we noted that some mutations (15.5%) can be identified in both sample types which is called the shared mutation (Fig. 1D). Besides, only a few private mutations (7.6%) can be detected in plasma. In a very large proportion of BM patients, CSF can provide more unique genetic mutations (76.9%) than plasma (7.6%). Hence, CSF identification of BM mutation spectrum is more advantageous.

**Fig. 1:**
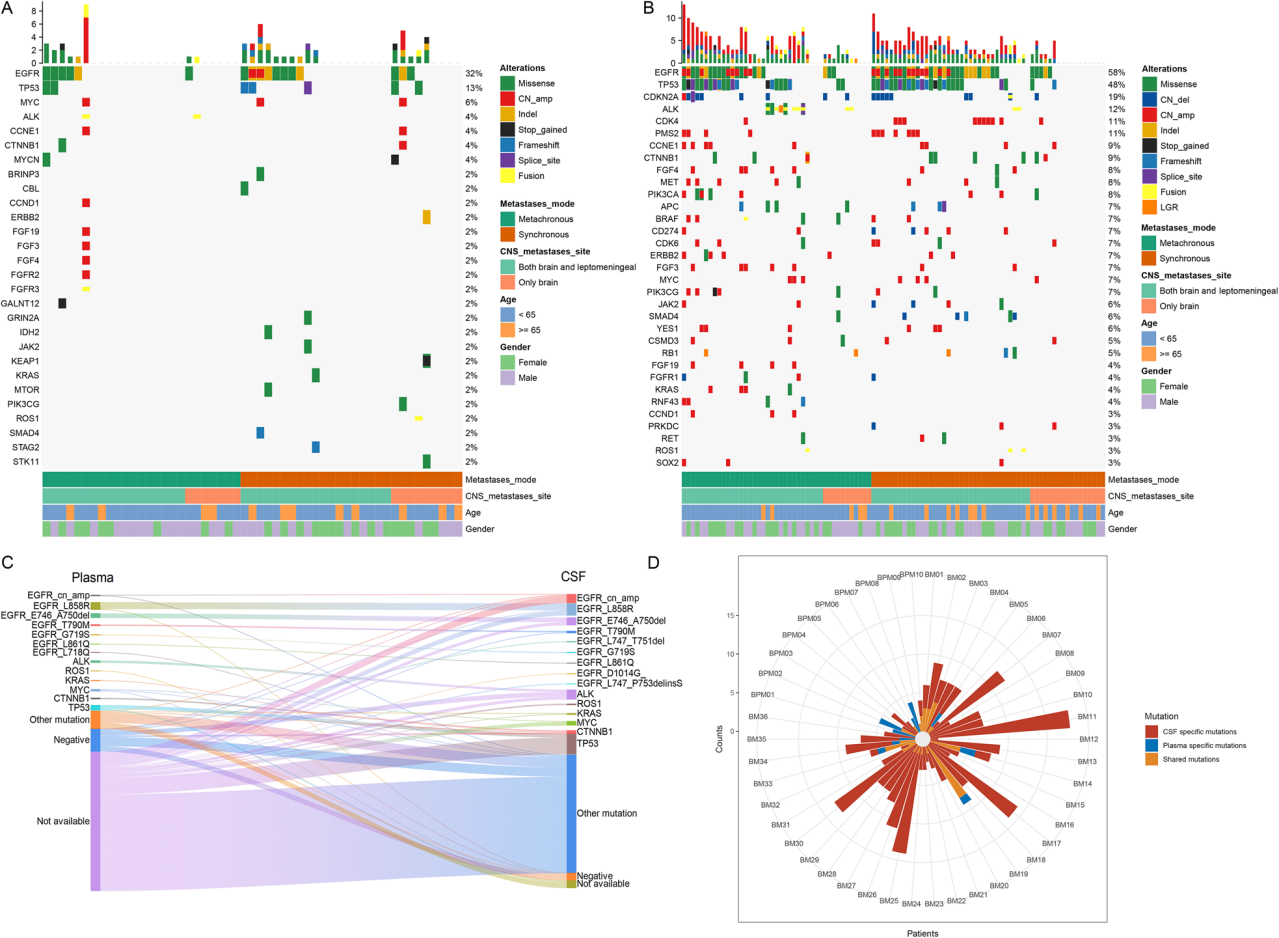
Overview of the mutational landscape of the plasma and CSF liquid biopsies from patients. **A**. Mutation profiles of the most frequently detected genes in the plasma samples, including SNV (single nucleotide variants), CNV (copy number variants), and other alterations. Each column represents a patient, each row represents a gene, and the corresponding mutation rates are provided on the right side. Altered gene information and clinical characteristics are annotated in the right panel. The upper panel showed the number of altered genes. **B** Mutation profiles of the high mutated genes in the CSF samples **C**. Discordance of detected altered genes in paired CSF and plasma. cn, copy number; amp, amplification. Negative, genes not be detected in one sample but could be detected in the other kind of sample, or not detected in both samples. Not available, genes not be detected in one sample but detected in the other kind sample; **D**. Gene counts of shared, CSF-specific, or plasma-specific alteration in paired CASF-plasma samples. CSF, cerebrospinal fluid; BPM, brain parenchymal metastases. BM, brain parenchymal metastases and leptomeningeal metastases

Subsequently, we removed copy number alterations and extracted the information on single nucleotide variations to perform the further analysis. We focused on the molecular features of altered somatic mutations between synchronous and metachronous metastases. The most common mutations were *EGFR* and *TP53*. SBM patients harbor more mutation in *CTNNB1* while *APC* and *ALK* were more mutated genes in the MBM group (Fig. 2A). Variant allele frequency (VAF) in the synchronous metastatic tumor showed high dissimilarity to those in the metachronous metastatic tumor. And, we noted that *EGFR* and *TP53* have a high allelic frequency in the metachronous group (Fig. 2B). Next, we characterize the molecular profile of variants in *EGFR* and *TP53*, In the SBM cohort, the most common EGFR mutation type is exon 19 deletion, while in the MBM cohort, L858R point mutation is the most frequent EGFR mutation type (Fig. 2C). Therefore, for synchronous brain metastasis patients with the 19 deletion mutation, we recommend the first-line use of osimertinib. For non-synchronous brain metastasis patients with L858R mutation, the combination of bevacizumab and erlotinib could be considered as a first option. Four missense TP53 mutations were found in SBM patients, including D74, S116, D153 and T377. R273 was a unique TP53 mutation site in MBM patients (Fig. 2D). Then, we performed gene enrichment analysis to identify enriched signaling pathways. Mutated genes of both cohorts were enriched in RTK-RAS and TP53 pathways. NFR2 accounted for a large fraction of affected pathways in the MBM group (Fig. 2E and F). In addition, we have interrogated the pharmacogenomic interactions in both groups to explore potential druggable medicine. The clinically actionable genes in the MBM group were *ALK*, *APC*, *ARID1A*, *AXIN2*, *BRAF*, while *APC*, *ATRX*, *CBL*, *CDK6*, *CTNNB1* were the clinically actionable genes for the SBM group. The distinct drug-gene associations also revealed the heterogeneous nature of the two cohorts (Additional file 5: Figure S5).

**Fig. 2:**
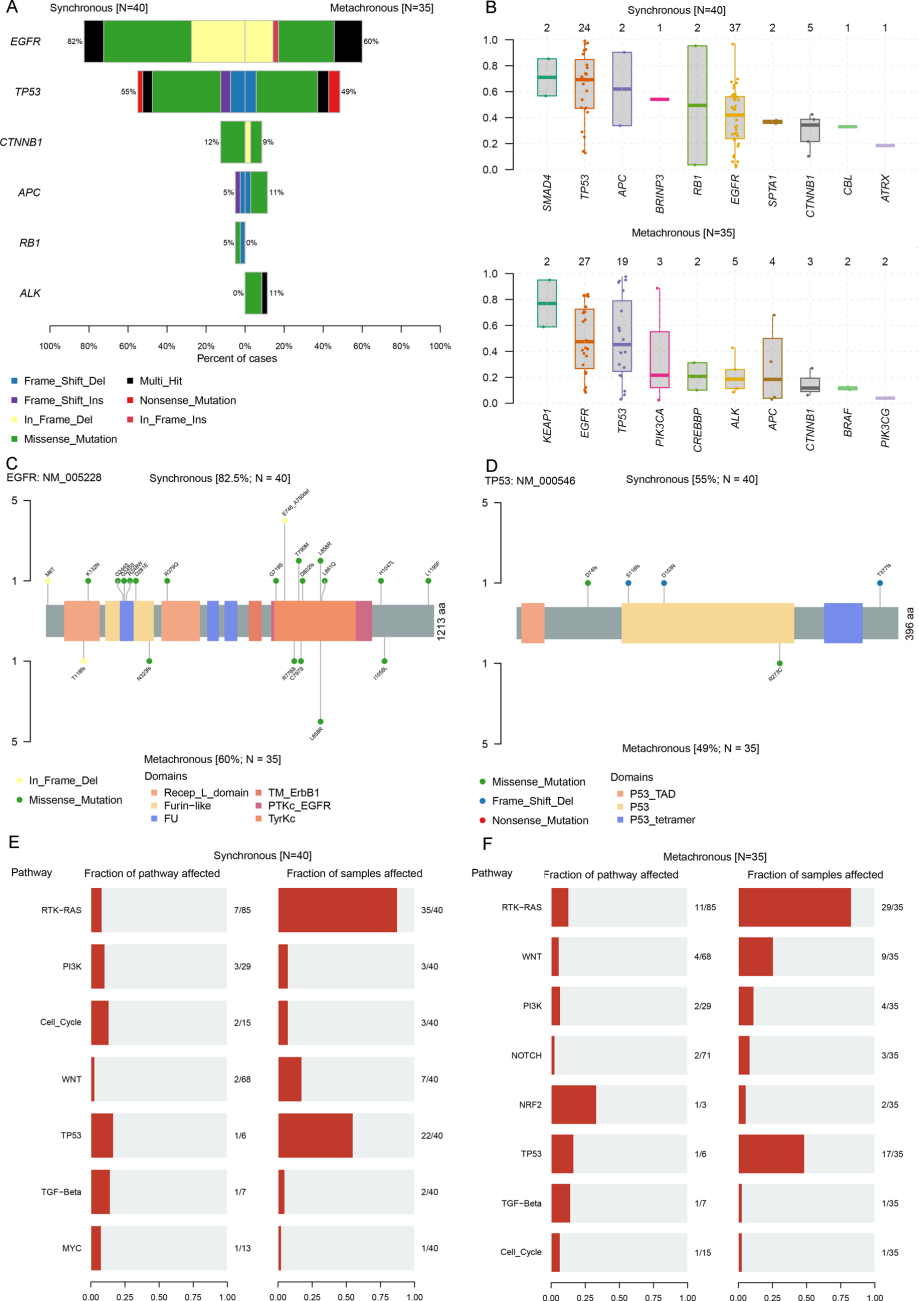
CSF cfDNA molecular characterization of patients with SBM and MBM. **A**. Comparison of significantly different altered genes (FDR < 0.05) between the SBM cohort (left) and MBM cohort (right); **B**. The variant allele frequencies of frequently altered genes in both cohorts; **C**. The summaries of EGFR mutation locations; **D**. The summaries of TP53 mutation locations; **E**. The fraction of affected pathways and samples in the SBM cohort; **F**. The fraction of affected pathways and samples in the MBM cohort

Altogether, our study strengthened the rationale for the use of CSF to detect clinical drive alterations of brain parenchymal metastases. The comparison of genomic characterization revealed the similarities and disparities between synchronous and metachronous brain metastases. These potentially clinically actionable targets present in the brain metastases are critical for the development of more effective treatment strategies to improve outcomes for these two distinct populations.

## Supplementary Information

**Additional file 1: Figure S1. **Kaplan–Meier survival curves of OS for patients with brain metastases in metachronous and synchronous patterns.

**Additional file 2: Figure S2.** Kaplan–Meier survival curves of Brain overall survival for patients with brain metastases in metachronous and synchronous patterns.

**Additional file 3: Figure S3.** Mutation profiles of the most frequently detected genes in the plasma samples of BM patients combined with liver metastases.

**Additional file 4: Figure S4.** Mutation profiles of the most frequently detected genes in plasma samples from patients with brain metastases and concomitant bone metastases.

**Additional file 5: Figure S5.** The barplot of pharmacogenomic interactions analysis. Drug-gene interaction of synchronous metastases patientsand metachronous metastases patients

**Additional file 6: Table S1.** Clinical characteristics of brain metastases patients.

## Data Availability

The datasets analyzed for the current study are available from the corresponding author on reasonable request.

## Abbreviations

BM: Brain metastasis
BOS: Brain overall survival
LM: Leptomeningeal metastasis
CSF: Cerebrospinal fluid
SBM: Synchronous brain metastases
MBM: Metachronous brain metastases
OS: Overall survival
VAF: Variant allele frequency
