# Supplementary figures and images for: Mutational signatures of synchronous and metachronous brain metastases from lung adenocarcinoma
Source: Exp Hematol Oncol. 2023 Jun 13;12:54. doi: 10.1186/s40164-023-00418-x (PMC10265840; doi:10.1186/s40164-023-00418-x)

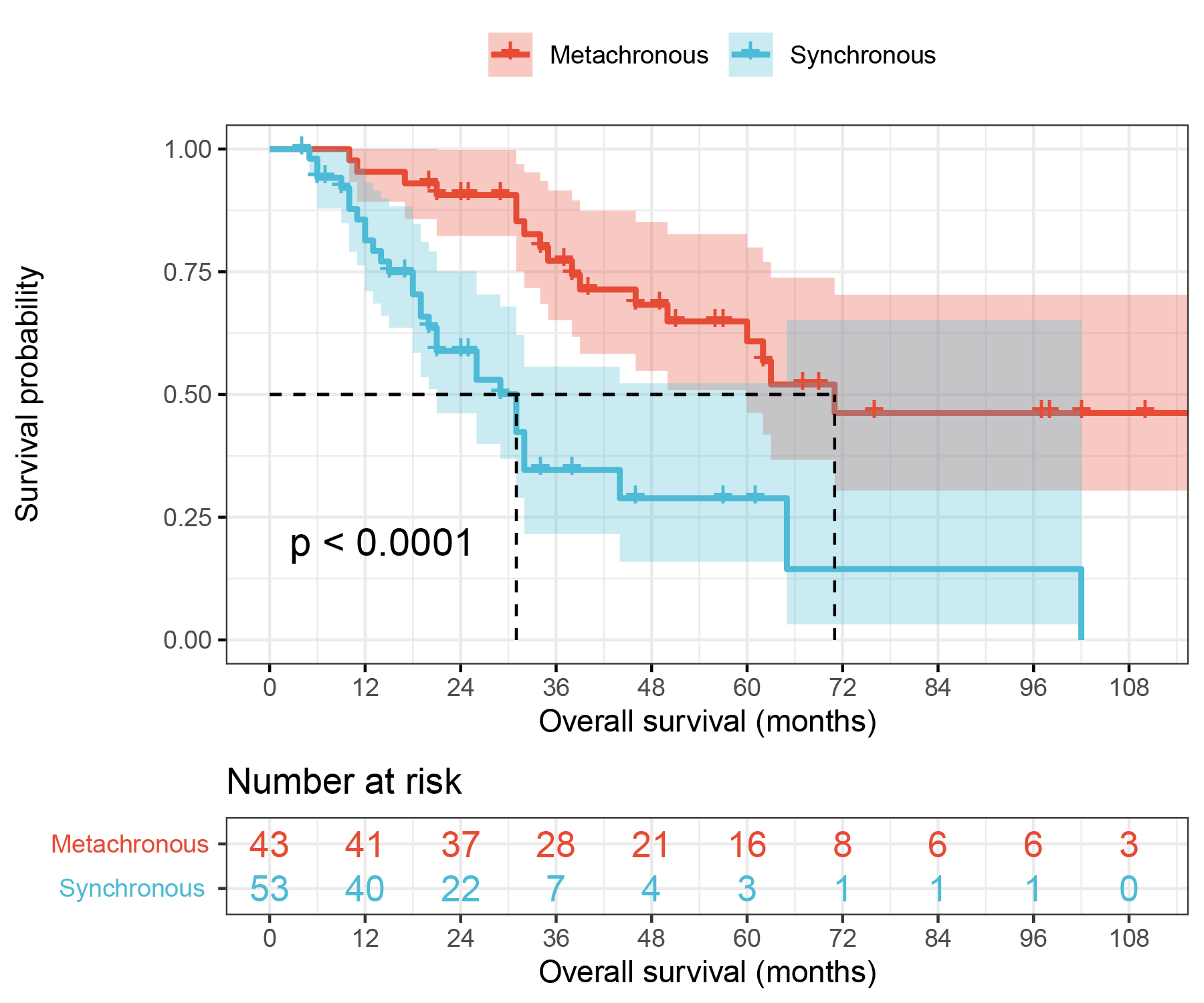

Supplement: Supplementary file 1 — Additional file 1: Figure S1. Kaplan–Meier survival curves of OS for patients with brain metastases in metachronous and synchronous patterns. [file 40164_2023_418_MOESM1_ESM.tif]

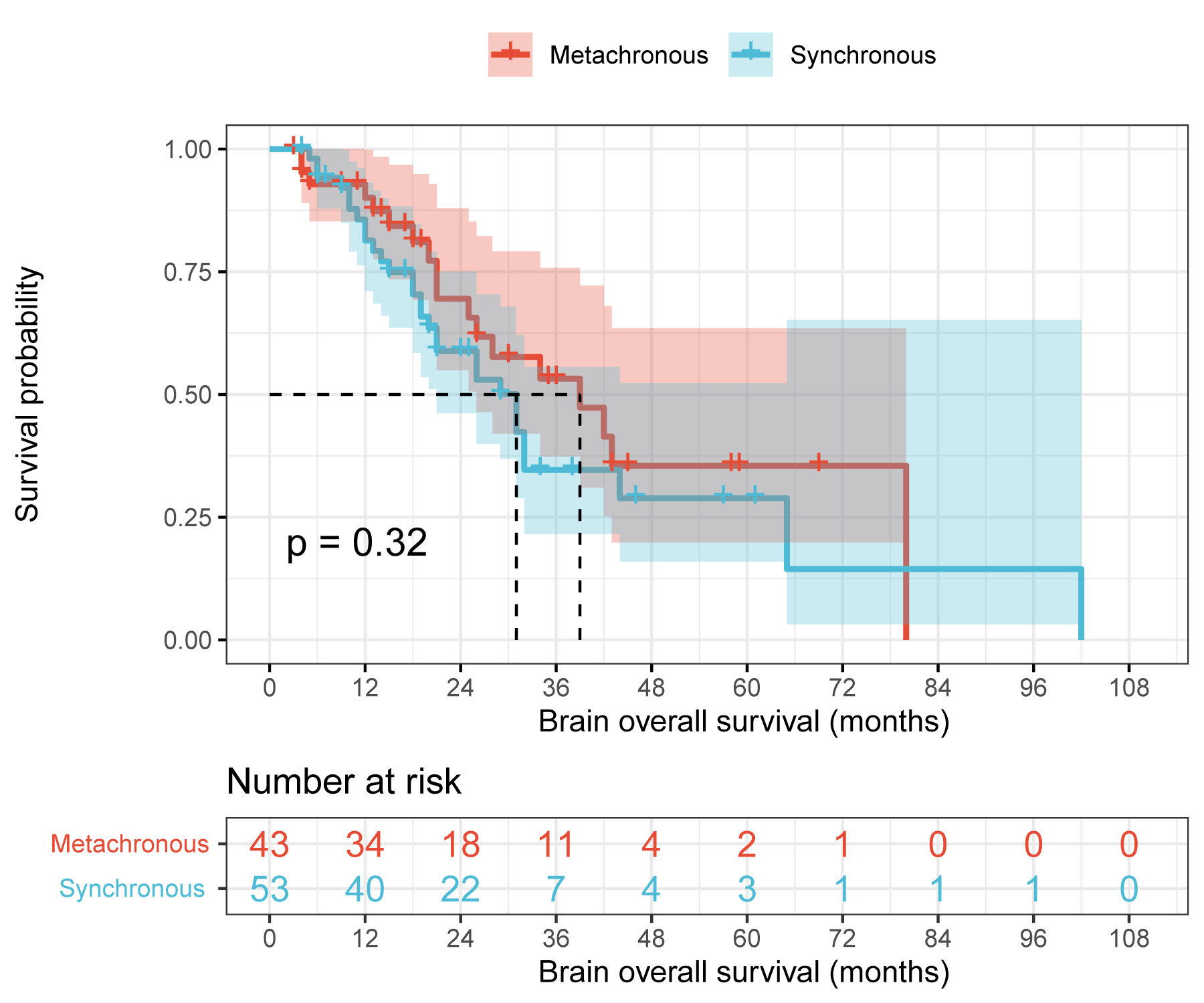

Supplement: Supplementary file 2 — Additional file 2: Figure S2. Kaplan–Meier survival curves of Brain overall survival for patients with brain metastases in metachronous and synchronous patterns. [file 40164_2023_418_MOESM2_ESM.tif]

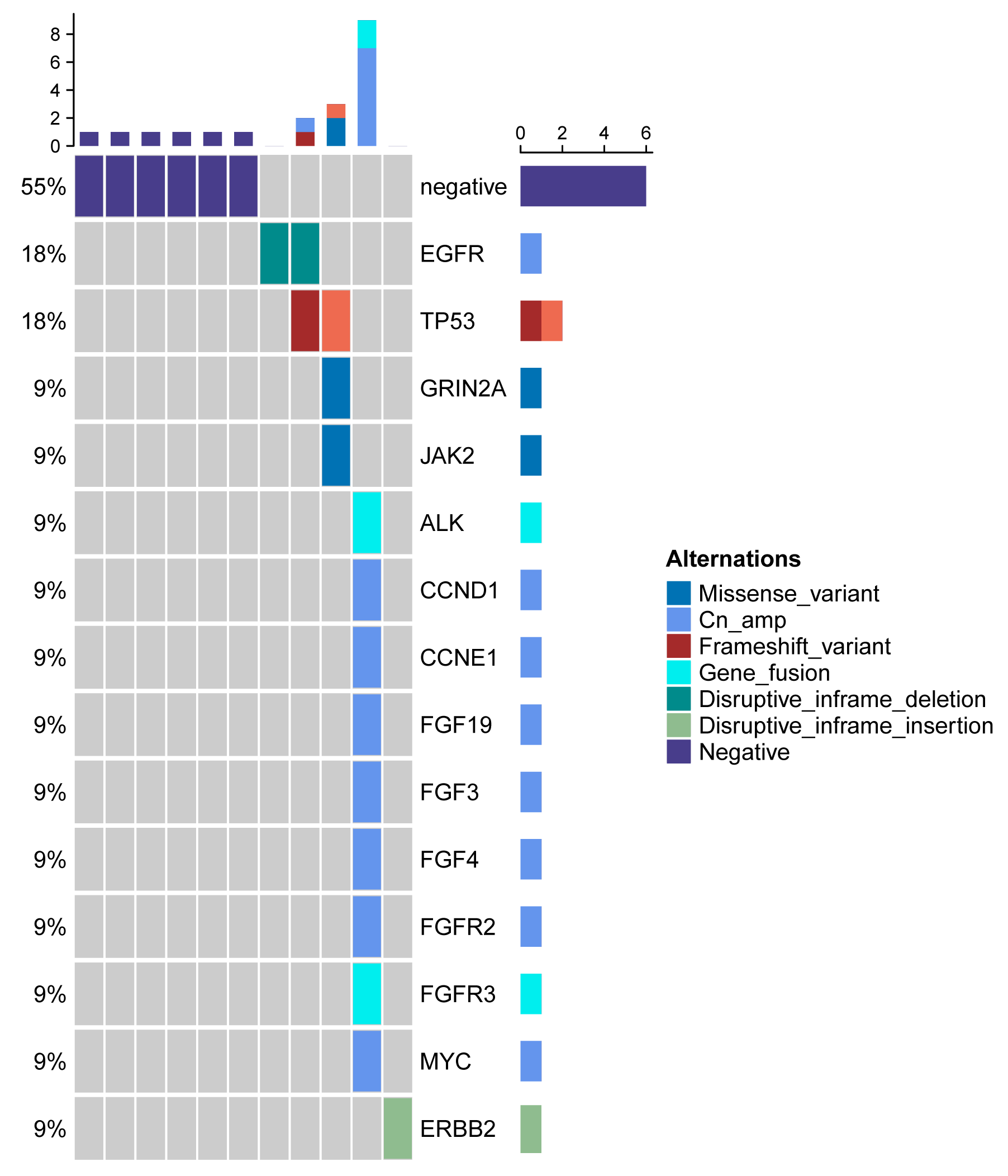

Supplement: Supplementary file 3 — Additional file 3: Figure S3. Mutation profiles of the most frequently detected genes in the plasma samples of BM patients combined with liver metastases. [file 40164_2023_418_MOESM3_ESM.tif]

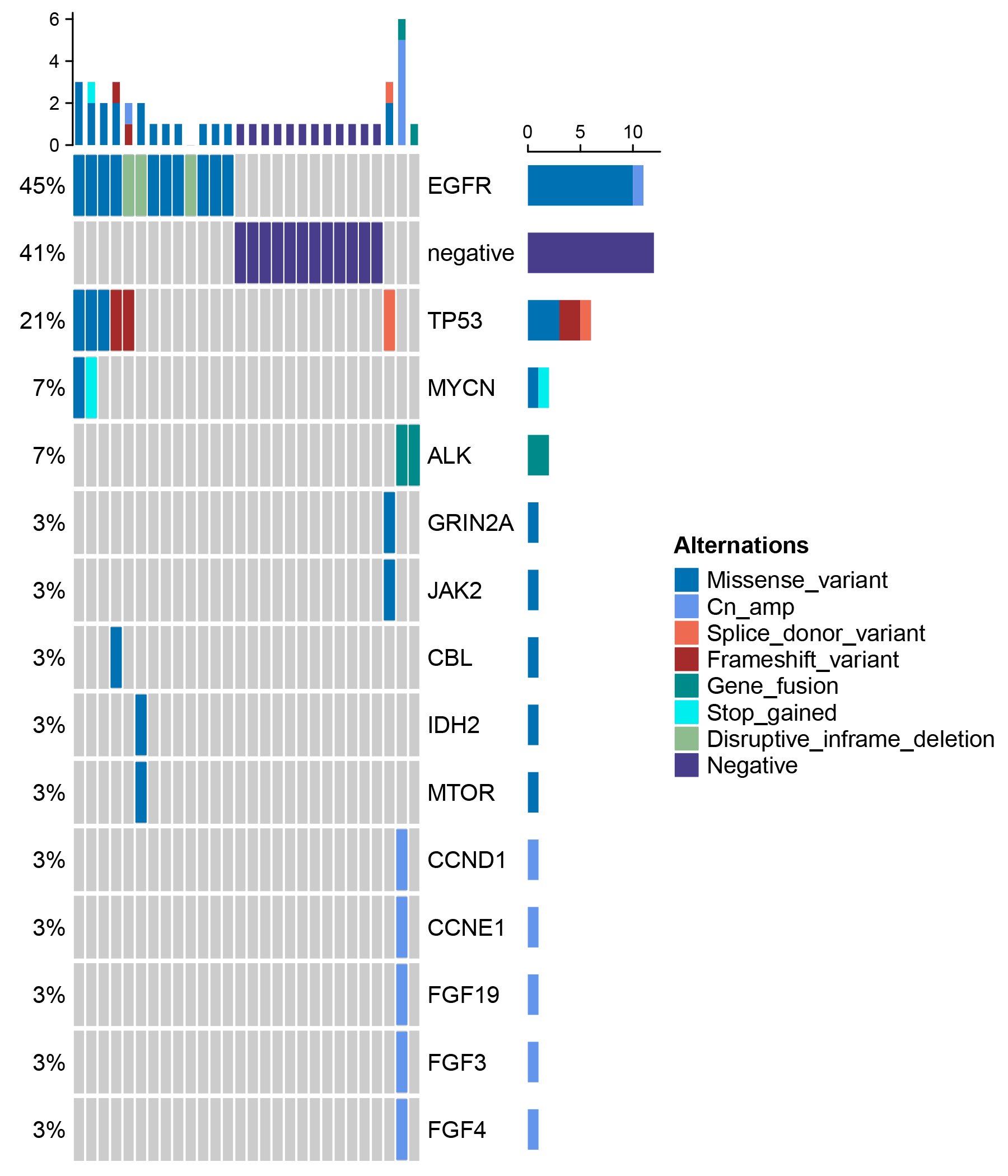

Supplement: Supplementary file 4 — Additional file 4: Figure S4. Mutation profiles of the most frequently detected genes in plasma samples from patients with brain metastases and concomitant bone metastases. [file 40164_2023_418_MOESM4_ESM.tif]

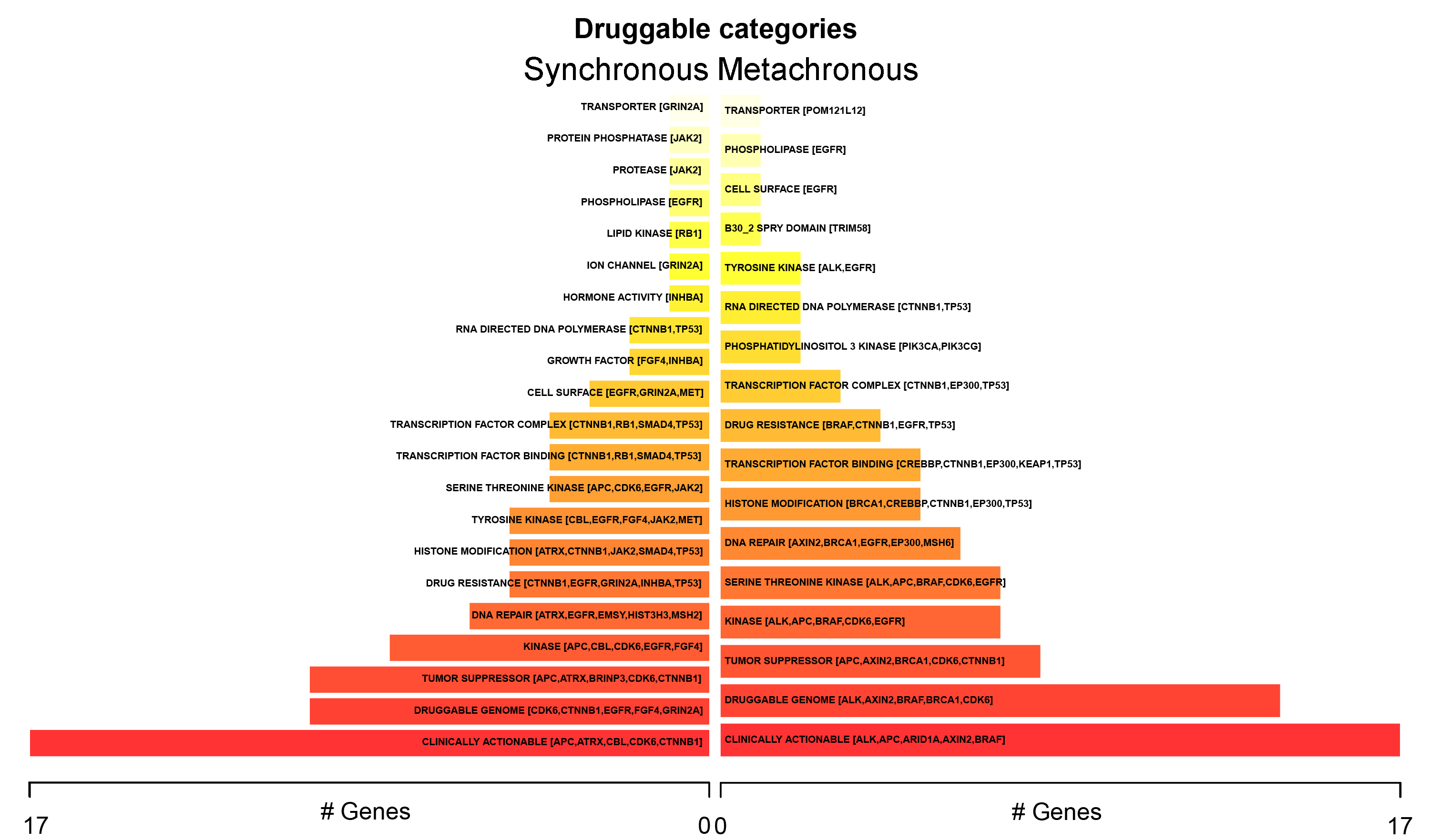

Supplement: Supplementary file 5 — Additional file 5: Figure S5. The barplot of pharmacogenomic interactions analysis. Drug-gene interaction of synchronous metastases patientsand metachronous metastases patients [file 40164_2023_418_MOESM5_ESM.tif]
