# Supplementary material for: Mutational signatures of synchronous and metachronous brain metastases from lung adenocarcinoma
Source: Exp Hematol Oncol. 2023 Jun 13;12:54. doi: 10.1186/s40164-023-00418-x (PMC10265840; doi:10.1186/s40164-023-00418-x)
Supplement: Supplementary file 6 — Additional file 6: Table S1. Clinical characteristics of brain metastases patients. [file 40164_2023_418_MOESM6_ESM.docx]

**Table 1** Clinical characteristics of brain metastases patients

| **Clinicopathological characteristics** | **Synchronous**  **(n=53)** | **Metachronous**  **(n=43)** | ***p*-value** |
| --- | --- | --- | --- |
| **Gender** |  |  | 0.68 |
| Male | 27(50.9) | 24(55.8) |  |
| Female | 26(49.1) | 19(44.2) |  |
| **Age, years** |  |  | 0.08 |
| ≥65 | 14(26.4) | 5(11.6) |  |
| <60 | 39(73.6) | 38(88.4) |  |
| **Histology** |  |  |  |
| Adenocarcinoma | 53(100.0) | 43(100.0) |  |
| The stage at initial diagnosis, No. (%) |  |  | <0.0001 |
| I-III | 0(0.0) | 20(46.5) |  |
| IV | 53(100.0) | 23(53.5) |  |
| **Gene mutation** |  |  | 0.33 |
| wild | 2(3.8) | 2(4.7) |  |
| EGFR | 21(39.6) | 15(34.8) |  |
| ALK | 2(3.8) | 7(16.3) |  |
| ROS1 | 2(3.8) | 1(2.3) |  |
| NA | 26(49.0) | 18((41.9) |  |
| **Site of metastases** |  |  | 0.51 |
| BPM | 17 | 11 |  |
| Both BPM and LM | 36 | 32 |  |

BM: Brain parenchymal metastases; LM: leptomeningeal metastases; The Chi-square test was used to compare the population proportions difference in gene mutation between the two groups; Fisher’s exact test was used to calculate the *p* values in other differences.
